# Effectiveness of personal protective equipment—Yes, the buck and virus can stop here

**DOI:** 10.1017/ice.2021.75

**Published:** 2021-02-19

**Authors:** Alan C. Howell, Lisa Havens, Wes Swinford, Alejandro C. Arroliga

**Affiliations:** 1 Division of Infectious Disease, Department of Internal Medicine, Baylor Scott and White Health, Temple, Texas; 2 Risk Management, Baylor Scott and White Health, Temple, Texas; 3 Baylor Scott and White, Temple, Texas

*To the Editor—*Coronavirus disease 2019 (COVID-19) has adversely affected the health and well-being of our communities as well as our places of work in countless ways. Healthcare providers (HCPs) across the country continue to step forward in support of their communities and families. These workers are a critical yet finite resource. Thus, it is important they and the systems in which they work take the appropriate steps to prevent severe acute respiratory coronavirus virus 2 (SARS-CoV-2) exposure and COVID-19. Herein, we discuss our experience as a healthcare system regarding the effectiveness of personal protective equipment (PPE) and the sources of exposure.

Baylor Scott and White Health (BSWH) is the largest not-for-profit healthcare system in Texas and one of the largest in the United States. Our system includes 52 hospitals, 7,300 active physicians, and ˜42,000 employees. Since the pandemic began, system recommendations regarding the use and type of PPE have remained consistent with guidance provided by the Centers for Disease Control and Prevention (CDC). Additionally, BSWH set up an Employee Health COVID Command Center (EHCCC) in early 2020. The purpose of the EHCCC was to have 1 centralized contact for employees with questions about exposures, travel, symptoms, testing, medical leave, and pay. Both patient and community encounters were reported to the EHCCC. The EHCCC created and maintained a single database for monitoring trends and developing reports. Other responsibilities of the EHCCC included fit testing as well as managing the exemption requests and return-to-work issues to maintain safe work environments for staff and patients. EHCCC collaborated with infection control and clinical leaders on providing guidance and developing protocols.

As of August 31, 2020, 12,405 employees had had direct exposure to SARS-CoV-2–positive patients due to their job duties. Moreover, 1,639 of these employees were tested for SARS-CoV-2 based upon the nature of their exposure and/or onset of symptoms consistent with COVID-19. Testing was performed using a nasal swab and a nucleic acid amplification test (NAAT). Overall, 87 employees tested positive. Our observed conversion rate was 0.70% by encounter and 0.20% by total staff. The median time from COVID-19 patient exposure to positive employee test was 11 days. Finally, between March and August, the greatest number of positive SARS-CoV-2 tests occurred in April, when 54 employees tested positive. Thereafter, the number of positive tests dropped precipitously, and for July and August, the number of positive tests were 3 and 1, respectively.

During this same period, 7,486 employees reported community exposures. 3,990 of these employees were tested due to their described exposure and/or onset of COVID-19 symptoms. Moreover, 1,136 tested positive for SARS-CoV-2, for a conversion rate of 15.17% by encounter and 2.67% by total staff. The number of positive tests per month for employees with reported community exposure peaked in July (478 employees tested positive). June, July, and August accounted for ∼93% of the 1,136 positive tests collected from March through August.

A lack of adequate PPE, work overload, insufficient diagnostic testing, and exposure to infected patients are all factors linked to the risk of infection for healthcare personnel.^1^ Our rate of work-related exposure compares favorably to the 6.3% rate noted at the Cleveland VA Medical Center.^1^ The effectiveness of PPE is highly dependent upon appropriate staff training, adherence to strict hand hygiene, and appropriate human behavior.^2^ This latter point carries significant importance regardless of whether HCP are carrying out their employment-related duties or are in the community. The fact that our conversion rate in the hospital setting was 0.70% versus 15.17% in the community supports this concept.

Importantly, no efforts are fail proof. As the pandemic continues, PPE and mask fatigue will set in. Hospital systems across the country are beginning to note evidence of this fact.^3^ The next struggle will be to determine strategies to best combat “battle fatigue.”

In summary, our experience with PPE, its availability, and our system approach (EHCCC) clearly demonstrate effectiveness. The risk community exposure poses to our finite number of HCP and patients remains.
